# On the Constitutional Treatment of Female Diseases

**Published:** 1857-06

**Authors:** 


ART. IV.—*On the Constitutional Treatment of Female Diseases.* By EDWARD RIGBY, M.,D., etc., etc., Fellow of the Royal College of Physicians; Senior Physician to the General Lying-in-Hospital; Examiner in Midwifery at the University of London. 12mo. pp. 256. Philadelphia: Blanchard & Lea. 1857.

The author of the above volume introduces his book by stating that his "observations on the uterine and other female affections have no claim to bring a complete work on the diseases of women. I do not offer it as a class book to those who are entering the medical profession, and whose studies must necessarily be chiefly, if not entirely, devoted to preparing themselves for their different examinations; but I venture to offer it, more especially to that large portion of my professional brethren, who, being engaged in general practice, have not been able to devote their special attention to a class of affections which nevertheless must frequently come under their notice. The work is strictly *practical;* and I have endeavored throughout, as far as I could, to have this in view."

Notwithstanding the modest disclaimer in the above introduction, we apprehend that but few persons will read this little volume, without arriving at the conclusion that the writer was actuated by higher aspirations for the honors of authorship than are therein disclosed.

The book was very evidently written in a spirit of antagonism to the largely prevalent views of the day relative to the influence exerted by the uterus over the health of the female, localizing in this organ the origin of that multiude of ills which embitter the lives of so many women. Dr. Rigby finds cause for all these difficulties elsewhere, and makes the uterus secondary in the chain of causes, or rather he scarce dignifies this organ with so important a position, but makes it the passive sufferer of ills engendered elsewhere in the system. With him everything is *constitutional*, and we cannot divest ourselves of the impression that he desires credit for originality of discovery of the importance of constitutional treatment in these diseases. Certain it is that he ignores local causation and local treatment so perfectly, that no one from what he teaches could conceive either to be of the least importance. There is an assumption in the title of the book which we dislike. The value of constitutional treatment is not denied by the most urgent advocates of local treatment. If they render less necessary a long course of administration of drugs, by recognizing and addressing their remedies to local disease, it argues but little discernment in the practitioner to preach of constitutional disease and treatment as the sum of medical knowledge upon these matters. We dislike, moreover, these attempts to block out distinct modes of practice. A treatise should contain, and should give the *treatment* of whatever form of disease may be under discussion, unless it be for the purpose of advocating some new or improved system of practice, and which, from its design, does not propose to enter into the discussion of the subject in all its bearings. That such is the character of the work before us we do not admit. *Constitutional treatment* is not new with Dr. Rigby, and he inculcates in his book but one mode of practice for all his diseases.

The etiology and pathology of this book are decidedly *hepatic*. Torpid liver, torper and derangement of the chylopoietic viscera, confined bowels, loaded intestines are the evil genii which stand guard over on the avenues to health, and are to be courted and mollified ere they will withdraw and allow the approach of more welcome visitors.

The remedial measures are in keeping with the etiology. Calomel, blue pills, and colycinth, are the remedies for all the troubles; whatever else may be used are but accessories. So prominent a place is given to mercurials that it recalls to mind the old days of the triumph and glories of the mineral, and seems really a step backward in the pathway of progress.

The deductions from such simple premises are plain. What we have been accustomed to regard as so many distinct and specific diseases are results and developments of the malign influences exerted by the derangements in the economy from the causes above specified. We will give our author the privilege of speaking for himself:

"I have, therefore, devoted what may appear an unusual amount of consideration to the functional derangements, particular by those of menstruation; not only because they are affections of every day occurrence, but because I am particularly anxious their close connection with the general health, and its various conditions, especially as regards the chylopoietic viscera, should be thorougly understood; being convinced that on a right apprecia-tion of this, depends not only their correct diagnosis, but also the principles of their sneccessful treatment.

"I have devoted separate chapters to the consideration of these subjects (Amenorrhœa, Dysmenorrhœa, &c.) solely in deference to a long established custom, which in former times was a necessity, when their nature and essen-tial causes were imperfectly or erroneously understood, and which has been sanctioncd by force of habit; but I feel assured that my readers will agree with me in the conviction that the time will come when those terms, as well that of leucorrhora, will no longer designate distinct affections, but will be classed with such symptoms as pain, rigors, expectoration, &c.

"Neither do I consider that organic disease of the female generative organs is to stand as an exception to the importance of constitutional treat-ment; for I look upon it (to use an admirable expression of Dr. Latham's, on pulmonary consumption) as "no more than a *fragment* of a constitu-tional malady!"

These views pervade the whole book and of course color the descriptions of causations and modes of treatment. The reader will undoubtedly turn the most directly to the chapters on the "Inflammation of the Os and Cervix Uteri," and "Ulceration of the Os and Cervix Uteri," for the purpose of obtaining the author's views upon these two subjects, as they form prominent themes of discussion at the present time. These subjects, of so much im-portance, are treated in two short chapters.

We make the following quotation from the chapter treating of the first form of disease:

"In treating the subject of the inflammation of the os and cervix uteri, I must beg it to b[illegible] remembered that I do not feel justified in looking upon it commonly as a prim[illegible]ry idiopathic disease, but rather as one which is of a secondary character; [illegible] in other words, symptomatic of some cause, the presence of which has inauced it. I can no more look upon inflammation of the os and cervix uteri as a primary disease, causing derangement of the general health, &c., than I could on a gouty toe, a rheumatic knee-joint, or enlarged strumous gland. Most of these uterine affections are the local manifestations of some general derangement, but which, in turn, react as causes, producing their own set of sympathies and effects."

Accordingly we find, that inflammation of the os and cervix uteri seldom occurs as an acute affection, but, in far the majority of cases in a subacute or chronic form; is usually associated with considerable derangement of the digestive organs, and by an atonic condition of the system generally; and is caused by whatever tends to produce or keep up uterine congestion. "Constipated bowels or torpid liver are two of the most fiequent causes of this affection." He admits that inflammation of the cervix may be induced by causes of a more local character, exposure to cold, for instance, but this is more liable to produce inflammation of the ovaries, especially if acting during a catamenial period. He adds one other cause, "the dishonest application of caustic to the os uteri."

In reference to treatment, he says in view of "the amount of general derangement of health which attends a case of ordinary inflammation of the os and cervix uteri, it is highly desirable to premise some doses of alterative and laxative medicine, and thus thoroughly to clear out the bowels, &c., before proceeding to any special local remedies. A dose of blue pills for two or three successive nights, and a brisk laxative the following mornings, frequently produce such a change in the general symptoms as to materially alter the severity of the uterine affection. By restoring the liver to a healthy action, and clearing the intestines of a large quantity of feculent matter, the abdominal circulation becomes frequently relieved, and local congestion proportionably diminished. If the os uteri still remains much congested, and of the dark red color already alluded to, it is better at once to scarify the part, as being the quickest and most effective mode of relieving the patient." Leeches are recommended in cases of a more chronic character.

In cases of longer standing, and which are decidedly of a chronic character, with induration of the part, with severe pain, and uterine congestion, the confession is extorted that:

"The general treatment is by no means so simple or so easy as in the other case. The health has been so much deranged by the long continued effects of severe uterine irritation, and the strength so broken down by frequent attacks of menorrhagia, and the profuse leucorrhœa during the intervals, that it is difficult to adopt any distinct line of treatment at first starting, beyond the attempt to regulate the liver and bowels by the mildest remedies, and soothe the irritable system by gentle sedatives."

Pray, of what use is a *constitutional* course of treatmeut if it cannot, and is not to reach such a class of cases as these. Calomel, blue pills, and colycinth here fail indeed. The author very briefly points out a more rational course of treatment of alteratives and tonics, which we should conclude would be calculated to reach the constitutional troubles, if anything could.

"Ulceration of the Os and Cervix Uteri," is treated in a still shorter manner.

He charges, "that the subject of ulceration of the os and cervix uteri has been of late years exaggerated to a remarkable extent, both as regards its supposed frequency and its effects, and presents an instance of delusion as discreditable to the candor of a practitioner as to the common sense of his patients. It has been asserted that about nine out of ten patients owe their various symptoms to this cause; and to support such an assertion, every variety of appearance which the os uteri presents, as seen through a speculum, has been pronounced to be "ulceration," although, in reality, having nothing whatever to do with it; and numerous cases have come under my own personal knowledge where caustic has been even applied to a perfectly healthy os uteri, and the patient informed that she had "ulceration!"

"Ulceration of the os and cervix uteri, not connected with malignant disease of the uterus, is in fact, a rare affection. It depends essentially upon passive congestion of the organ—as in prolapsus, and is especially observed after abortion, where the health has been much broken and the uterus has remained large and flabby, and where from general debility and want of tone, it has continued lower in the pelvis than natural. In other cases, this congested state has evidently been produced by constipation, torpid liver, and other derangements of the chylopoietic viscera."

The *general* treatment of these difficulties must be conducted on the same rules as in inflammation of the os and cervix uteri. It is somewhat amusing to observe, that after the denunciatory manner he has spoken of those practioners, who favor the application of caustics in the treatment of these ulcerations, that he is at last compelled to prescribe them in his plan of treatment. He says, "where there is a less amount of inflammatory action, the application of solid lunar caustic instantly stops the ulcerative process, by destroying the unhealthy granulations, and covering the part with an eschar, under which a healthier process becomes established."

Again, further on he says, "If these objects have been attained, and the case be one of recent date, a single *free* application of caustic not only produces no irritation of the part, but after a short time, great relief; the darting pains, the hardness, swelling and tenderness, are much diminished and the discharge becomes more healthy."

We have referred to these two chapters at this length, for they evidently contain the gist of the book. They, however, add but little to our stock of knowledge of these diseases. The chapter on "Inflammation of the Os and Cervix Uteri" is lacking in clearness of style. The description of the disease is confusedly written, and it is impossible at times to know whether he is describing the disease itself, or the factitious symptoms which he asserts are produced by the "dishonest" use of caustics. The same ambiguity attends upon his description of the mode of treatment. The readiness with which he imputes dishonesty of motive to the advocates of local medication, adds nothing to the pleasure of the reader.

The discussion of the subjects of "Displacements of the Uterus," "Retroversion," "Anteversion," "Prolapsus Vesicæ," "Polypus Uteri," interferes with the uniformity of the *hepatic* pathology, and these complications are treated in the usual manner, although the usual changes are rung on the necessity of regulating the functions of the chylopoietic organs.

The chapter on the "Fibrous Tumor of the Uterus" approaches nearer to the proper treatment of the subject than the sections which precedes it. After a description of the tumour, drawn principally from Mr. Paget, reference is made to the different methods proposed for getting rid of these bodies by a process of absorption.

He gives a case occurring under his own observation where a tumour disappeared under the effects of repeated leechings. He has observed beneficial results follow the continued use of the chloride of calcium. Bromide of potassium, added to an artificial preparation of Kreuznach water, he has used with apparent advantage.

Had a greater portion of the book been written with the same spirit which characterizes the treatment of this subject, a much more valuable volume would have been the result.

"Malignant Diseases of the Uterus" are treated at considerable length, without, however, much originality being displayed in their consideration. In treatment of cancer, iron is extolled, especially the sulphate. Arsenic is also favorably alluded to, and the *iodide of arsenic* preferred. Upon the benefits attending the administration of the latter compound, Dr. Walshe is quoted.

A consideration of the subjects of "Pruritus Pudendi," "Vascular Tumor of the Orifice of the Urethra," "Ovarritis," "Displacement of the Ovary," and "Ovarian Tumors," closes the book, but with very little attracting especial attention, except it be one subject, which as it seems to be the most, if not the only *original* contribution to the volume, we will notice more at length.

Under the head of "Displacements of the Ovary," the author says:

"Of late years, I have had occasion to notice a form of ovarian displacement which, as far as I am aware of, has not been hitherto described, and which, on account of the intense suffering it produces, as also the character of its diagnosis and mode of its treatment, is of great practical importance. I allude to where the ovary descends into the rectovaginal pouch, and occupies a position between the rectum and the uterus, and almost justifying the term *prolapsus of the ovary*."

The displacement is characterized by intense and peculiarly sickening pain about the sacral region, extending to one or other of the groins, coming on in paroxysms of agonizing severity, and excited by the smallest pressure, as by the passages of the fœces. The menstrual periods are always attended with greatly increased suffering, particularly during the early part of the discharge. The real position is detected by a careful examination by the vagina and rectum.

He speaks of seeing several cases, but the account is imperfect in not stating the number, and the comparative frequency of the accident. These are both points of interest which should not have been omitted.

In concluding our notice of this volume, we are compelled to say after a careful perusal of it, that we cannot see how it can add to the reputation of the author. It is true the treatment is sufficiently orthodox to come within the pale of regular practice, but it is not in keeping with the advancements of medical science. It lacks originality of investigation both in theory and practice, and has too much the evidence that it was written, because its author felt that it was necessary for him to write a book for the increase, or preservation of present reputation. Its partisan spirit is the only exhibition of sufficient life it presents, to relieve it from unmitigated dullness. N.

